# Praktisches Management der Sjögren-Erkrankung im Zeitalter neuer Therapieoptionen: Aktueller Stellenwert der verfügbaren Outcome-Parameter

**DOI:** 10.1007/s00393-026-01799-0

**Published:** 2026-04-10

**Authors:** Jürgen Braun, T. Dörner

**Affiliations:** 1 Rheumatologisches Versorgungszentrum Steglitz, Schlossstr. 110, 12163 Berlin, Deutschland; 2 Ruhr Universität Bochum, Bochum, Deutschland; 3 Medizinische Klinik mit Schwerpunkt Rheumatologie und Klinische Immunologie, Charité – Universitätsmedizin Berlin, Campus Charité Mitte Charitéplatz 1, 10117 Berlin, Deutschland; 4 DRFZ Berlin, Berlin, Deutschland

**Keywords:** Sicca-Syndrom, Speicheldrüsenentzündung, Sjögren-Erkrankung, Sjögren’s Syndrome Disease Activity Index, Sjögren’s Syndrome Patient Reported Index, Sicca syndrome, Sialadenitis, Sjögren’s disease, Sjögren’s Syndrome Disease Activity Index, Sjögren’s Syndrome Patient Reported Index

## Abstract

Die Sjögren-Erkrankung (Sjögren’s Disease, SjD) ist eine chronisch-entzündliche Autoimmunerkrankung, von der überwiegend Frauen betroffen sind, die vor allem unter Trockenheit der Schleimhäute, insbesondere im Augen- und Mundbereich, Müdigkeit und potentiell auch extraglandulären Manifestationen wie Gelenk- und Muskelschmerzen leiden. Bei der initialen Diagnostik spielen neben der Klinik serologische Veränderungen, speziell Autoantikörper gegen SS-A(Ro)/SS-B(La) sowie Rheumafaktoren eine wichtige Rolle. Die Pathogenese ist zwar noch nicht abschließend geklärt, von einer bedeutenden Rolle von B‑Zellen ist aber auszugehen. Bisher gab es vor allem symptomatische Therapiemöglichkeiten, die glandulären Funktionsdefizite bezüglich der Sicca-Symptomatik zu behandeln. Basierend auf den letzten Publikationen besteht berechtigte Hoffnung, dass sich dies bezüglich der extraglandulären Manifestationen bald ändern könnte. Dafür ist es besonders wichtig, dass validierte Outcome-Parameter zur Verfügung stehen, mit denen der mögliche Erfolg von neuen Therapien evaluiert werden kann. In diesem Beitrag werden die von der EULAR (European Alliance of Associations for Rheumatology) bereitgestellten Werkzeuge EULAR Sjögren’s Syndrome Disease Activity Index (ESSDAI) zur Erfassung der extraglandulären und Sjögren’s Syndrome Patient Reported Index (ESSPRI) bzgl. der patientenberichteten Outcomes (PRO) übersetzt und im Detail vorgestellt. Beide stellen auch die Grundpfeiler möglicher Outcome-Instrumente (STAR bzw. CRESS) der 2. Generation, die derzeit validiert werden, dar.

## Abstract (en)

Sjögren’s disease is a chronic inflammatory autoimmune disease, which mostly affects women who suffer particularly from dryness of mucous membranes, especially in the eyes and the mouth, tiredness and potentially also extraglandular manifestations such as joint and muscle pain. In the initial diagnostic work up serological alterations especially autoantibodies against SS-A(Ro)/SS-B(La) and rheumatoid factors play an important role. The pathogenesis is not yet finally clarified but an important role of B cells can be assumed. To date mostly symptomatic treatment options to improve glandular functional deficits with respect to the sicca symptoms are available. Based on most recent publications there is justifiable hope that this could soon change, especially with respect to extraglandular manifestations. For this reason, it is particularly important that validated outcome parameters are available to enable proper evaluation of new treatment options. In this article the EULAR instruments Sjögren’s syndrome disease activity index (ESSDAI) for documenting extraglandular manifestations and Sjögren’s syndrome patient reported index (ESSPRI) as patient-reported outcome (PRO) have been translated and are here presented in detail. Both also represent the cornerstones of possible outcome instruments (STAR or CRESS) of the second generation, which are currently undergoing validation.

Das vormals sog. Sjögren-Syndrom, nun aktualisiert als Sjögren-Erkrankung (SjD) bezeichnet [1], ist eine chronisch-entzündliche Autoimmunerkrankung, bei der zelluläre und humorale Autoimmunprozesse vor allem gegen sekretbildende Körperdrüsen gerichtet sind; dabei kommt es zu verschiedenen extraglandulären Manifestationen [2]. Die Pathogenese dieser erstmals 1933 vom schwedischen Augenarzt Henrik Sjögren beschriebenen Erkrankung ist noch nicht eindeutig geklärt. Die SjD kann als eigenständige Erkrankung primär oder in Assoziation mit anderen Autoimmunerkrankungen, wie z. B. der rheumatoiden Arthritis oder dem systemischen Lupus, auftreten. Die lange geläufige Bezeichnung sekundäres Sjögren-Syndrom wurde zuletzt von Experten aufgegeben [1].

Das Leitsymptom bei Patientinnen mit Sjögren-Syndrom ist eine anhaltende Mund- und Augentrockenheit (Sicca-Symptomatik), die in über 95 % der Fälle bei Frauen auftritt [2]. Diese Bevorzugung von Frauen konnte bislang nicht hinreichend geklärt werden.

Die betroffenen Patientinnen klagen häufig über starke Erschöpfung und andere Symptome wie Gelenk- und Muskelbeschwerden, welche die Lebensqualität erheblich beeinträchtigen können. Darüber hinaus kann es zu geschwollenen Speicheldrüsen und Lymphknoten, Hautveränderungen und Durchblutungsstörungen in den Fingern (Raynaud-Syndrom), kommen. In seltenen Fällen können darüber hinaus Lunge, Nieren, Blutgefäße und/oder das periphere (PNS) oder zentrale Nervensystem (ZNS) geschädigt werden [2].

Die Zulassung des Anti-CD20-Antikörpers Rituximab zur Behandlung von mittelschwerer bis schwerer rheumatoider Arthritis (RA) bei Patienten, die nicht auf Anti-Tumornekrosefaktor(TNF)-Wirkstoffe ansprechen [3], hat seit dem Jahr 2006 das Interesse an einer B‑Zell-gerichteten Therapie zunehmend nicht nur für diese Erkrankung geweckt, zumal wenig Zweifel daran besteht, dass auch bei anderen entzündlich-rheumatischen Erkrankungen wie dem Lupus erythematodes und der SjD B‑Zellen von pathogenetischer Bedeutung sind [4, 5]. Klinische Erfahrungen nicht nur mit Rituximab legen nahe, dass die gezielte Bekämpfung von potenziell autoimmunen Gedächtnis-B-Zellen ein praktikabler Ansatz zur Behandlung von bestimmten Autoimmunerkrankungen wie Lupus, SjD und auch dermatologischen und neurologischen Autoimmunerkrankungen ist [6]. Allerdings haben bisherige Studien zur Anti-CD20-Depletion mit Rituximab oder Zytokininhibition (BAFF [B-Zell-aktivierender Faktor], TNF‑α, Lymphotoxin-β) keine hinreichende Differenzierung der Wirkstoffe zum Kontrollarm erbracht. Demgegenüber stehen erfolgreiche Studien von Pilokarpin, Civemelin und Ciclosporin-A-haltigen Augentropfen zur lokalen Kontrolle der Entzündung und Funktionsverbesserung der Drüsenproduktion [7, 8].

Die Behandlung der extraglandulären SjD hat sich in den letzten Jahrzehnten nicht wesentlich verändert. Somit bleiben Behandlungsentscheidungen in der klinischen Praxis eine Herausforderung, da es außer der symptomatischen Symptomlinderung bisher kein spezifisches und erreichbares therapeutisches Ziel gegeben hat. So haben sich auch die letzten Empfehlungen der EULAR (European Alliance of Associations for Rheumatology; [6]) für das Management der SjD auf die Behandlung der drei zentralen Symptome Trockenheit, Müdigkeit (Fatigue) und Schmerzen konzentriert. Die systemische Therapie der SjD orientiert sich insbesondere an Erfahrungen bei anderen rheumatischen Erkrankungen wie der rheumatoiden Arthritis (RA). Entsprechend beziehen sich die Empfehlungen vor allem auf die Anwendung topischer oraler Speichelersatzmittel und okulärer Therapeutika wie künstliche Tränenflüssigkeit, topische nichtsteroidale Entzündungshemmer, topische Kortikosteroide, und topisches Ciclosporin A sowie orale Muskarinagonisten wie Pilocarpin und Cevimelin [7, 8]. Systemische Therapien wie Hydroxychloroquin, orale Glukokortikoide, synthetische Immunsuppressiva (Cyclophosphamid, Azathioprin, Methotrexat, Leflunomid und Mycophenolat) und biologische Therapien (Rituximab, Abatacept und Belimumab) werden durchaus [9–11], partiell auch mit Erfolg, eingesetzt, sind aber nicht für die SjD zugelassen [12].

So ist in den letzten beiden Jahrzehnten eine Reihe direkter und indirekter Modalitäten mit unterschiedlichen Wirkmechanismen untersucht worden, darunter Anti-CD20- und Inhibitoren der CD40-CD40-Ligand(L)-Interaktionen sowie neue Ansätze zur Blockierung von Signalwegen der TNF-Superfamilie, dem BAFF-Rezeptor (BAFF-R) und dem Zytokin „another proliferation-inducing ligand“ (APRIL), die sich in einem fortgeschrittenen Stadium der klinischen Entwicklung der SjD befinden [13–17].

Eine wichtige Herausforderung im rheumatologischen Alltag ist die Messung der Krankheitsaktivität

Eine wichtige klinische Problematik und Herausforderung im rheumatologischen Alltag ist die Messung der Krankheitsaktivität der SjD [18]. Hierzu gibt es wesentliche Aktivitäten der EULAR Task Force, die dazu Vorschläge erarbeitet und weiter evaluiert hat [19–24]. Leider stehen weder der EULAR Sjögren’s Syndrome (SS) Disease Activity Index (ESSDAI) noch der EULAR Sjögren’s Syndrome Patient Reported Index (ESSPRI) bis jetzt in einer deutschen Version zur Verfügung, was wahrscheinlich auch daran liegt, dass es bisher keine systemischen Therapien gab, die eine standardisierte Messung erforderlich gemacht hätten. Da sich das möglicherweise bald ändert, haben wir hier wichtige Daten der Originalpublikationen übersetzt und zusammengefasst. Der Vollständigkeit halber muss erwähnt werden, dass beide Outcome-Instrumente zentrale Säulen neuer Messinstrumente der 2. Generation Sjögren’s Tool for Assessing Response (STAR) bzw. Composite of Relevant Endpoints for Sjögren’s Syndrome (CRESS) sind, die derzeit evaluiert werden [25, 26]. Hintergrund ist die Zusammenführung der ESSDAI- und ESSPRI-Komponenten in differenzierter Wertigkeit und Hinzunahme der Sicca-Komponenten und Patient’s Global Assessment (PAGA) als Composite Scores.

## Methoden

Die Originalpublikationen von ESSDAI [19] und ESSPRI [20] wurden zunächst von JB übersetzt, dann von TD überprüft und, wenn erforderlich, geändert.

### EULAR Sjögren-Syndrom-Krankheitsaktivitätsindex (ESSDAI)

Der *ESSDAI* [19] umfasst insgesamt 12 Domänen zur Erfassung der extraglandulären Organmanifestationen (Tab. 1). Zum Verständnis und zur Anwendung ist wichtig, dass jede Organmanifestation eine bestimmte Wichtung (Wertigkeit) erhält. Innerhalb dieser werden unterschiedliche Schweregrade definiert. Jede ESSDAI-Domäne enthält 3–4 Schweregrade („disease activity levels: no – low – moderate – high“) zwischen 0 und 3 (je Domäne) und wird mit der Organwichtung der Domäne multipliziert, um in den Gesamt-ESSDAI-Score einzugehen.

Zur Elimination von rein laborbedingten Veränderungen, die klinisch nicht relevante Veränderungen betreffen können, wurde der *clinESSDAI *eingeführt [21]. Dieser eliminiert nur die biologische (die 12.) Komponente. Theoretisch kann der maximale ESSDAI-Score also 123 Punkte umfassen. Eine Verbesserung von mindestens 3 Punkten wurde in einer internationalen Validationsstudie als signifikant definiert [22].

**Tab. 1:** Domänen des ESSDAI (EULAR Sjögren’s Syndrome Disease Activity Index), ihre individuelle Wichtung und ihr Beitrag zum Gesamt-ESDDAI

Domäne	Gewicht	Wichtung der Scores			
*Aktivitätslevel*		Keine (0)	Gering (1)	Moderat (2)	Hoch (3)
Biologisch	1	0	1	2	n. a.
Gelenkbezogen	2	0	2	4	6
Hämatologisch	2	0	2	4	6
Glandulär	2	0	2	4	n. a.
Haut	3	0	3	6	9
Konstitutionell	3	0	3	6	n. a.
Lymphadenopathie	4	0	4	8	12
Nierenbeteiligung	5	0	5	10	15
Lungenbeteiligung	5	0	5	10	15
Peripheres Nervensystem	5	0	5	10	15
Zentralnervensystem	5	0	n. a.	10	15
Muskulär	6	0	6	12	18
Summe				0–123 (maximal)	

Moderate Krankheitsaktivität: ESSDAI ≥ 5–8

Hohe Krankheitsaktivität: ESDAI ≥ 14

## Übersetzung der 12 Domänen des ESSDAI und organspezifische Aktivitätsbewertung im Einzelnen

Vorauszuschicken ist, dass die einzelnen Aktivitäten eindeutig der SjD zuzuordnen sind. Dies schließt andere Ursachen als auch präexistenten Schaden (irreversibler Funktionsverlust aus) aus.

### Originalpublikation ESSDAI [19]

Seror R, Ravaud P, Bowman SJ, et al.; EULAR Sjögren’s Task Force. EULAR Sjogren’s syndrome disease activity index: development of a consensus systemic disease activity index for primary Sjogren’s syndrome. Ann Rheum Dis 2010 Jun; 69 [6]:1103‑9. Epub 2009 Jun 28. Erratum in: Ann Rheum Dis 2011 May; 70 [5]:880. https://doi.org/10.1136/ard.2009.110619.

### Gebrauchsanleitung ESSDAI [23]

Seror R, Bowman SJ, Brito-Zeron P, Theander E, Bootsma H, Tzioufas A, et al. EULAR Sjögren’s syndrome disease activity index (ESSDAI): a user guide. *RMD Open*. 2015;1:e000022. https://doi.org/10.1136/rmdopen-2014-000022.

**Abb. 1:**
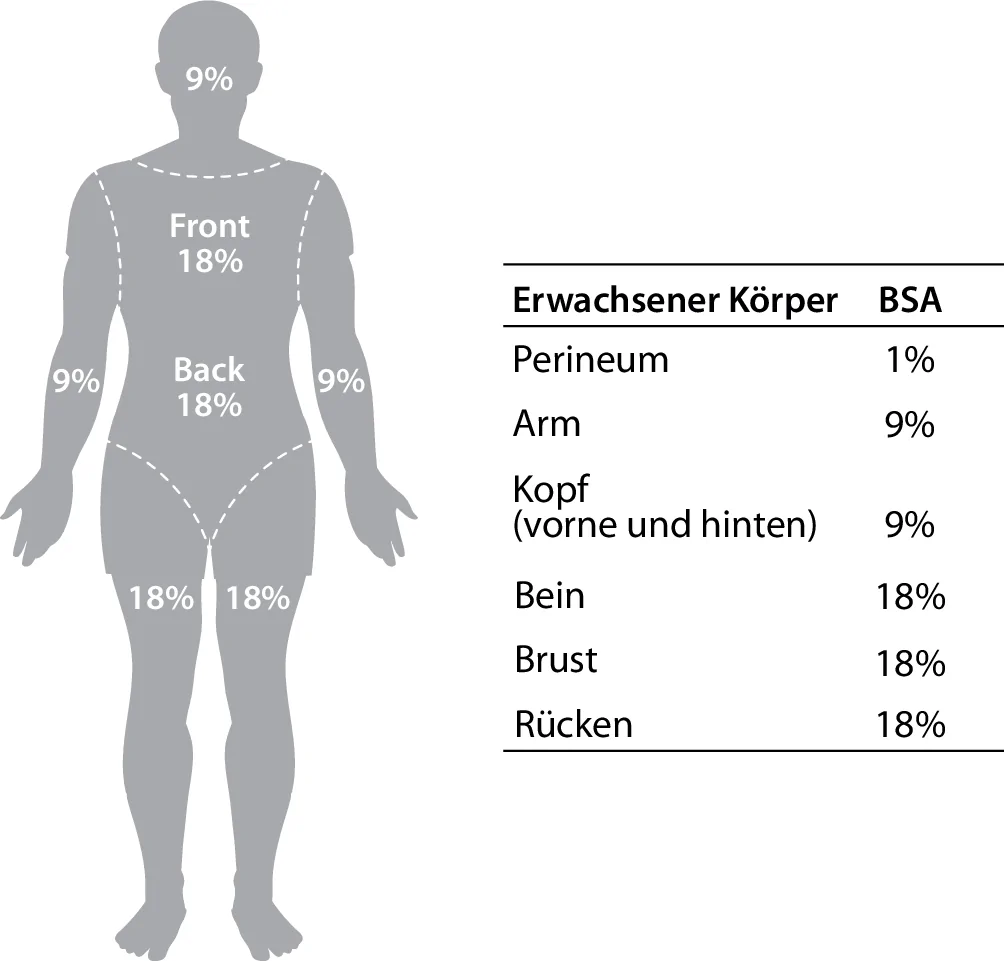
„Body surface area“ (BSA)

1. Konstitutionell (Ausschluss von Fieber durch eine Infektion und kein freiwilliger Gewichtsverlust)
  *Keine Aktivität:* keines der folgenden Symptome
  *Geringe Aktivität (3):* Fieber 37,5^o^–38,5^o^, Nachtschweiß und/oder Gewichtsabnahme 5–10 % des Körpergewichts
  *Moderate Aktivität (2):* Fieber > 38,5^o^, Nachtschweiß und/oder Gewichtsabnahme > 10 % des Körpergewichts
2. Lymphadenopathie (Ausschluss Infektion)
  *Keine Aktivität:* keines der folgenden Symptome
  *Geringe Aktivität (4):* Lymphadenopathie mit Lymphknoten ≥ 1 cm in irgendeiner Lymphknoten(LK)-Region oder ≥ 2 cm inguinal
  *Moderate Aktivität (8):* Lymphadenopathie mit Lymphknoten ≥ 2 cm in irgendeiner LK-Region oder ≥ 3 cm inguinal und/oder Splenomegalie (klinisch oder bildgebend)
  *Schwere Aktivität (12):* aktuelle maligne, proliferative B‑Zell-Erkrankung, Lymphom
3. Speicheldrüsen (Ausschluss von Lithiasis und Infektion)
  *Keine Aktivität (0):* keine Speicheldrüsenschwellung
  *Geringe Aktivität (2):* geringe Speicheldrüsenschwellung, mit vergrößerter Gl. parotis (≤ 3 cm), oder limitierter submandibulärer (≤ 2 cm) Schwellung oder Schwellung der Tränendrüsen (≤ 1 cm)
  *Moderate Aktivität (4):* erhebliche Speicheldrüsenschwellung mit vergrößerter Gl. parotis (> 3 cm), oder bedeutender submandibulärer (> 2 cm) oder Schwellung der Tränendrüsen (> 1 cm)
4. Gelenke (ohne Arthrose- oder sonstig bedingte Gelenkbeschwerden)
  *Keine Aktivität:* aktuell keine aktive Gelenkbeteiligung
  *Geringe Aktivität (2):* Arthralgien in Händen, Handgelenken, Knöcheln, Füße, begleitet von Morgensteifigkeit (> 30 min)
  *Moderate Aktivität (4):* Synovitis, 1–5 geschwollene Gelenke (von 28 Gelenken)
  *Hohe Aktivität (6):* Synovitis, ≥ 6 geschwollene Gelenke (von 28 Gelenken)
5. Haut (ohne Langzeitschäden) (Abb. 1)
  *Keine Aktivität:* keine aktuell aktive Hautbeteiligung
  *Geringe Aktivität (3):* Erythema multiforma
  *Moderate Aktivität (6):* limitierte kutane Vaskulitis (< 18 % BSA), inklusive Urtikaria-Vaskulitis oder Purpura, limitiert auf Füße und Knöchel, oder subakut-kutaner Lupus
  *Hohe Aktivität (9):* diffuse kutane Vaskulitis (≥ 18 % BSA), inklusive Urtikaria-Vaskulitis, oder diffuse Purpura oder Ulzera bedingt durch Vaskulitis (*BSA* „body surface area“)

6. Lungenbeteiligung (Abwesenheit von anderen Ursachen wie chronisch-obstruktive Lungenerkrankung [COPD], Nikotin etc.)
  *Keine Aktivität:* keine aktuell aktive Lungenbeteiligung
  *Geringe Aktivität (5):* persistierender Husten durch bronchiale Beteiligung ohne röntgenologische Abnormalitäten oder Evidenz von röntgenologischen oder HRCT-Befunden hinsichtlich einer interstitiellen Lungenerkrankung: keine Luftnot und normaler Lungenfunktionstest
  *Moderate Aktivität (10):* begrenzte moderat aktive Lungenbeteiligung bei interstitieller Lungenerkrankung gemäß dem HRCT-Befund mit Luftnot bei Belastung (NYHA II) oder einem pathologischen Lungenfunktionstest mit einer Diffusionskapazität (DLco) < 70 % aber ≥ 40 % oder einer forcierten Vitalkapazität (FVC) < 80 % aber ≥ 60 %
  *Hohe Aktivität (15):* schwere Lungenbeteiligung bei interstitieller Lungenerkrankung gemäß dem HRCT-Befund mit Luftnot in Ruhe (NYHA III-IV) oder pathologischem Lungenfunktionstest mit einer Diffusionskapazität (DLco) < 40 % oder einer forcierten Vitalkapazität (FVC) < 60 % *(HRCT* hochauflösende Computertomographie, *NYHA* New York Heart Association)
7. Nierenbeteiligung (ohne Langzeitschäden, Biopsieergebnis wird höher bewertet)
  *Keine Aktivität:* keine aktuell aktive Nierenbeteiligung mit Proteinurie < 0,5 g/Tag, keine Hämaturie, keine Leukozyturie, keine Azidose bzw. keine schon länger persistierende Proteinurie wegen bereits eingetretener Schädigung durch die Erkrankung
  *Geringe Aktivität (5):* milde Nierenbeteiligung, begrenzt auf tubuläre Azidose ohne Einschränkung der Nierenfunktion (GFR > 60 ml/min) oder glomeruläre Beteiligung ohne wesentliche Proteinurie (0,5–1 g/Tag) und ohne Hämaturie oder Einschränkung der Nierenfunktion (GFR ≥ 60 ml/min)
  *Moderate Aktivität (10):* moderat aktive Nierenbeteiligung, z. B tubuläre Azidose mit Einschränkung der Nierenfunktion (GFR < 60 ml/min) oder glomerulärer Beteiligung mit Proteinurie (1–1,5 g/Tag), ohne Hämaturie oder Einschränkung der Nierenfunktion (GFR ≥ 60 ml/min), oder histologische Evidenz von extramembranöser Glomerulonephritis oder relevanten interstitiellen lymphoiden Infiltraten
  *Hohe Aktivität (15):* schwere Nierenbeteiligung mit glomerulärer Beteiligung mit Proteinurie > 1,5 g/Tag, oder Hämaturie oder erheblicher Funktionseinschränkung (GFR < 60 ml/min), oder histologischer Evidenz von proliferativer Glomerulonephritis oder Nierenbeteiligung durch Kryoglobulinämie (*GFR* glomeruläre Filtrationsrate)
8. Muskelbeteiligung (keine Muskelschwäche bedingt durch Glukokortikoide oder andere Ursachen; Berücksichtigung assoziierter SjD bei Myasthenia gravis, idiopathische inflammatorische Myositis)
  *Keine Aktivität:* keine aktuell aktive muskuläre Beteiligung
  *Geringe Aktivität (5):* milde Myositis gemäß Magnetresonanztomographie (MRT), Elektromyographie (EMG) oder Biopsie, ohne Muskelschwäche und Kreatinkinase(CK)-Werten ≤ 2 des Normalwerts (*N*)
  *Moderate Aktivität (10):* moderat aktive Muskelbeteiligung gemäß MRT-, EMG- oder Biopsiebefund mit geringer Muskelschwäche (4/5), oder leicht erhöhter CK ≤ 4 *N*
  *Hohe Aktivität (15):* sehr aktive, schwere Muskelbeteiligung gemäß MRT-, EMG- oder Biopsiebefund mit erheblicher Muskelschwäche (≤ 3/5) oder deutlich erhöhter CK > 4 *N*

Bewertung der Muskelkraft:

*0:* Keine sichtbare Muskelkontraktion
*1:* Sichtbare Muskelkontraktion ohne oder mit kaum erkennbarer Bewegung
*2:* Bewegung der Gliedmaßen, nicht gegen die Schwerkraft
*3:* Bewegung gegen die Schwerkraft, aber nicht gegen Widerstand
*4:* Bewegung gegen zumindest ein bisschen Widerstand durch den Untersuchenden
*5:* Volle Kraftentwicklung

9.: Periphere Nervenbeteiligung (ohne Polyneuropathie [PNP] anderer Ursache):
  - *Keine Aktivität:* keine aktuell aktive Beteiligung des peripheren Nervensystems
  - *Geringe Aktivität (5):* mild aktive Beteiligung des peripheren Nervensystems, z. B. rein sensorische axonale PNP entsprechend der Nervenleitgeschwindigkeit (NLG) oder Trigeminusneuralgie oder Small-fiber-Neuropathie
  - *Moderate Aktivität (10):* moderat aktive Nervenbeteiligung gemäß NLG, z. B. sensomotorische axonale PNP mit maximalem muskulärem Defizit (4/5), rein sensorischer PNP durch Kryoglobulinämie, Ganglionopathie mit milder bis moderater Ataxie, entzündlicher, demyelinisierender PNP (CIDP) mit geringer Funktionsbeeinträchtigung (Motordefizit maximal 4/5 oder nur geringe Ataxie) oder kraniale periphere Nervenbeteiligung (außer N. trigeminus)
  - *Hohe Aktivität (15):* schwere aktive Nervenbeteiligung gemäß NLG, z. B. sensomotorische axonale PNP mit muskulärem Defizit von ≤ 3/5, periphere Nervenbeteiligung durch Vaskulitis (Mononeuritis multiplex etc.), schwere Ataxie durch Ganglionopathie oder demyelinisierende PNP (CIDP) mit schwerer Funktionsbeeinträchtigung (Motordefizit ≤ 3/5) oder schwere Ataxie
10.: Zentrale Nervenbeteiligung (außer vorhandenen ZNS-Schäden anderer Ursache, Berücksichtigung Differenzialdiagnose [DD] assoziierte SjD bei multipler Sklerose [MS] und Neuromyelitis-optica-Spektrum-Erkrankungen [NMOSD])
  - *Keine Aktivität:* keine aktuell aktive zentralnervöse Beteiligung
  - *Moderate Aktivität (10):* moderat aktive Nervenbeteiligung, wie kraniale Neuropathie mit zentralem Ursprung, Optikusneuritis oder MS-ähnliches Syndrom mit rein sensorischen Veränderungen oder kognitiver Beeinträchtigung
  - *Hohe Aktivität (15):* schwere, hoch aktive ZNS-Beteiligung, wie zerebrale Vaskulitis mit zerebrovaskulären Ereignissen oder transienten ischämischen Attacken, Anfällen, transverser Myelitis, lymphozytärer Meningitis, MS-ähnliches Syndrom mit motorischen Defiziten
11.: Hämatologische Beteiligung (Ausschluss Mangel an Vitaminen/Eisen oder medikamentenassoziierte Nebenwirkungen)
  - *Keine Aktivität:* keine autoimmune hämatologische Beteiligung (Zytopenie)
  - *Geringe Aktivität (2):* milde hämatologische Beteiligung mit autoimmuner Zytopenie (> 1000 aber < 1500 Neutrophile/mm^3^) und/oder Anämie (Hämoglobin > 10 aber < 12 g/dl) und/oder Thrombopenie (> 100.000 aber < 150.000 Thrombozyten/mm^3^) und oder Lymphopenie (> 500 aber < 1000 Lymphozyten/mm^3^)
  - *Moderate Aktivität (4):* moderate hämatologische Beteiligung mit autoimmuner Zytopenie (≥ 500 aber ≤ 1000 Neutrophile/mm^3^) und/oder Anämie (Hämoglobin ≥ 8 aber ≤ 10 g/dl) und/oder Thrombopenie (≥ 50.000 aber ≤ 100.000 Thrombozyten/mm^3^) und oder Lymphopenie (≤ 500 Lymphozyten/mm^3^)
  - *Hohe Aktivität (6):* schwere hämatologische Beteiligung, mit autoimmuner Zytopenie (< 500 Neutrophile/mm^3^) und/oder Anämie (Hämoglobin < 8 g/dl) und/oder Thrombopenie (< 50.000 Thrombozyten/mm^3^)
12.: Biologische Domäne (nicht Teil des *clinESSDAI*)
  - *Keine Aktivität (0):* keine biologische Beteiligung (keine Komplementerniedrigung [C4 oder CH 50] und keine Hypergammaglobulinämie)
  - *Geringe Aktivität (1):* klonale Komponente oder erniedrigtes Komplement (C4 oder CH 50) und/oder Hypergammaglobulinämie oder IgG-Serumspiegel zwischen 16 und 20 g/l
  - *Moderate Aktivität (2):* Kryoglobulinämie und/oder Hypergammaglobulinämie oder IgG-Serumspiegel > 20 g/l oder Hypogammaglobulinämie mit IgG-Serumspiegel < 5 g/l

### Der EULAR Sjögren Syndrome Patient Reported Index (ESSPRI)

#### Originalpublikation ESSPRI [20]

Seror R, Ravaud P, Mariette X, et al.; EULAR Sjögren’s Task Force. EULAR Sjogren’s Syndrome Patient Reported Index (ESSPRI): development of a consensus patient index for primary Sjogren’s syndrome. Ann Rheum Dis 2011 Jun; 70 [6]: 968–72. Epub 2011 Feb 22, https://doi.org/10.1136/ard.2010.143743.

**Abb. 2:**
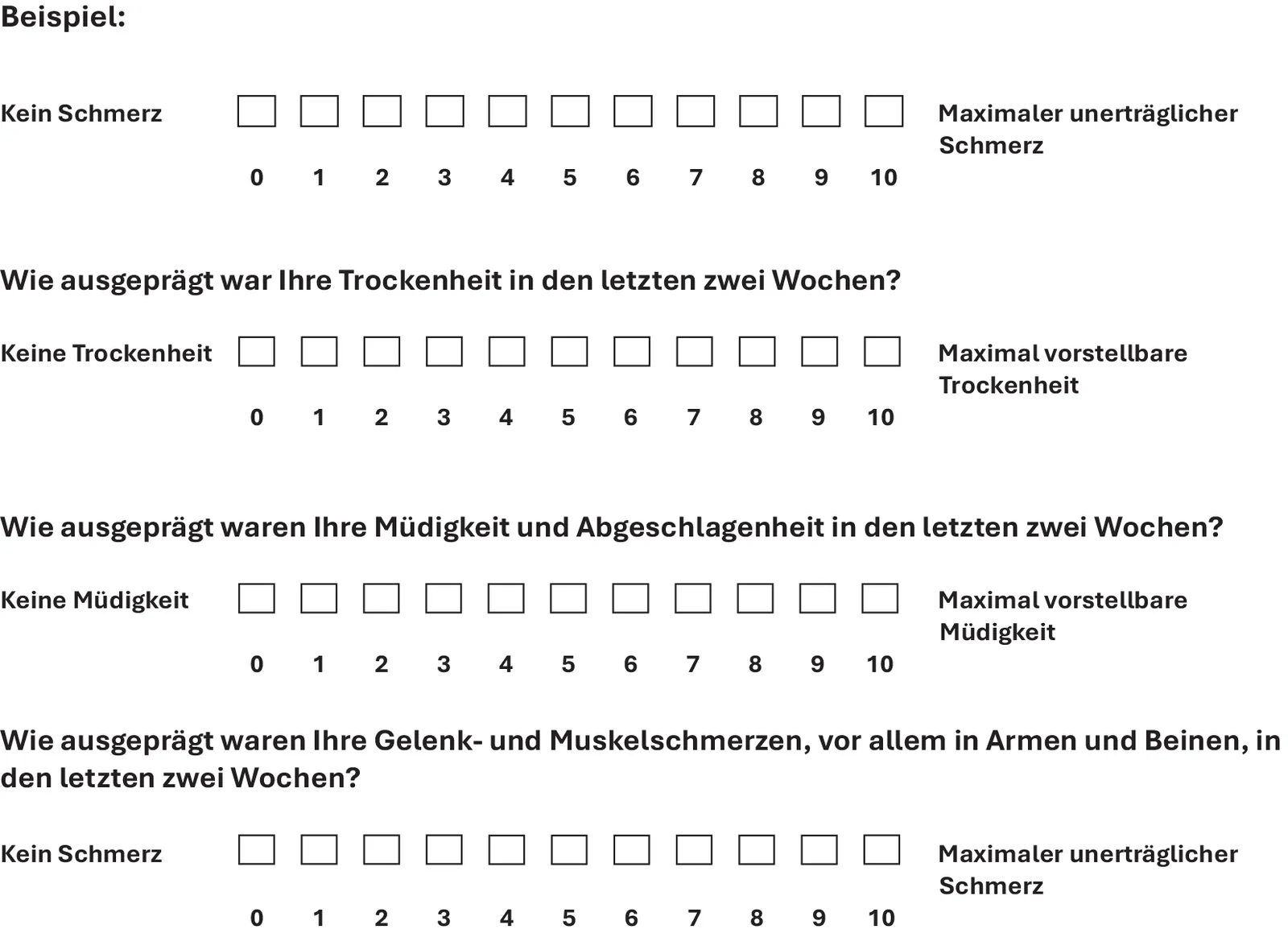
ESSPRI-Fragebogen für Patient*innen mit Sjögren-Syndrom (SjD). Bei der Beantwortung der Fragen sollen Sie bitte die Schwere Ihrer Symptome auf einer Skala zwischen 0 und 10 subjektiv, d. h. aus Ihrer Sicht, bewerten. Gesamtbeurteilung der letzten 2 Wochen. ([20]; Tab. 2 und 3)

Der *ESSPRI*-Fragebogen [20] ist ein typisches Patient-Reported Outcome (PRO), was aus 3 Punkten besteht, die mit einer Punktzahl zwischen 0 und 10 bewertet werden: Schmerzen (Gelenk- und/oder Muskelschmerzen), Müdigkeit und Trockenheit (0 = keine Symptome und 10 = schlimmste vorstellbare Symptome). Der Patient muss jeweils an der Stelle ankreuzen, die der Schwere seiner Symptome in den letzten 2 Wochen entspricht. Jeder Bereich repräsentiert die Schwere des Symptoms unabhängig voneinander; der arithmetische Durchschnitt der 3 Bereiche reflektiert den ESSPRI-Score (Abb. 2). Somit ergibt sich theoretisch ein Maximum im ESSPRI von 10. Eine klinisch relevante Verbesserung liegt bei einer Verminderung um 1 Punkt oder um 15 % im Score vor.

**Tab. 2:** Kombinierter Endpunkt [25] für das Therapieansprechen bei der Sjögren-Erkrankung (STAR)

Domäne		Punktzahl	Definition von Response
Systemische Aktivität	ESSDAI	3	≥ 3 clinESSDAI
PRO	ESSPRI	3	≥ 1 ESSPRI oder 15 %
Tränendrüsenfunktionstests	Schirmer-Test, Augen-Färbe-Score	1	≥ 5 mm mehr*
			≥ 2 Punkte weniger*
Speicheldrüsenfunktionstests	UWS, US	1	≥ 25 % Verbesserung des Flows* bzw. des Hocevar US-Score* gegenüber dem Ausgangsbefund
Biologische Aktivität	IgG, RF	1	Besserung ≥ 25 % bzw. ≥ 10 %
*STAR-Response*	**–**	**–**	≥ 5 Punkte

* bei normalem Ausgangsbefund keine Verschlechterung

*ESSDAI* EULAR Sjögren Syndrome Disease Activity Index, *ESSPRI* EULAR Sjögren Syndrome Patient Reported Index, *US* Ultraschall, *STAR* Sjögren’s Tool for Assessing Response

**Tab. 3:** Kombinierter Score [26] von relevanten Endpunkten für das Sjögren-Syndrom (CRESS)

Domäne	Systemische Krankheitsaktivität	Vom Patienten berichtete Symptome (PRO)	Tränendrüse	Speicheldrüse	Serologie
Methode	clinESSDAI	ESSPRI	Schirmer-Test, Ocular Staining Score	UWS, SGUS	RF, IgG
	Score < 5 Punkte = niedrige KH-Aktivität	Score ≥ 1 Punkt oder Verbesserung ≥ 15 % im Vergleich zum AgB	Wenn ST ≤ 5 mm, Steigerung ≥ 5 mm im Vergleich zum AgB	Steigerung ≥ 25 %	Abnahme ≥ 25 %
	–	–	Wenn OSS ≥ 3 Punkte, Abfall um ≥ 2 Punkte im Vergleich zum AgB	–	–
	–	–	Wenn beide initial normal sind, keine Verschlechterung	–	–

*AgB* Ausgangsbefund, *ST* Schirmer-Test, *OSS* Ocular Staining Score, *UWS* unstimulierte Speichelflussrate, *ESSDAI* EULAR Sjögren Syndrome Disease Activity Index, *ESSPRI* EULAR Sjögren Syndrome Patient Reported Index, *CRESS* Composite of Relevant Endpoints for Sjögren’s Syndrome

Die Durchflussmessung der unstimulierten Speichelflussrate (UWS) wird über einen Zeitraum von 15 min zu einer definierten Tageszeit, am besten vormittags nach einer dreistündigen Fastenzeit und ohne Tabakkonsum seit dem Vortag durchgeführt. Die Patienten werden gebeten, vor Beginn des Tests ihren Speichel zu schlucken und dann 15 min lang in einen Behälter zu spucken. Das Speichelvolumen wird nach dem Dekantieren mit einer auf 0,1 ml kalibrierten Spritze gemessen [27].

## Diskussion

Das wesentliche Ziel dieser Arbeit ist es, die SjD aufgrund absehbarer Entwicklungen für die Zulassung neuer Medikamente in den rheumatologischen Fokus zu rücken [11–14]. Das wird die Aufmerksamkeit auf diese nicht seltene rheumatische Erkrankung sehr wahrscheinlich erhöhen, obgleich die klinische Wirksamkeit der neuen Medikamente bei primärer und assoziierter SjD noch nicht abschließend geklärt ist. Ohne Zweifel sind standardisierte Messmethoden für Krankheitsaktivität und Verbesserung wie der ESSDAI und der ESSPRI [19–24] sowie die neuen Vorschläge für Outcome-Messungen [25, 26] von besonderer Bedeutung auch für das praktische Management.

In dieser Arbeit geht es zwar weniger um die Bewertung dieser Methoden als vielmehr um eine Übersetzung und Verfügbarkeit der Scores in der klinischen Anwendung, es sei jedoch angemerkt, dass der ESSDAI neben der Komplexität der einzelnen Organmanifestationen auch das methodische Problem eines sog. „floor effect“ beinhaltet. Ein solcher tritt auf, wenn ein Messinstrument oder Test zu umfangreich ist, wodurch eine große Anzahl von Personen niedrige Werte erzielt, was zu einer verzerrten Verteilung führt. Dadurch wird eine gute Präzision der Messungen verhindert, da die Unterschiede an der unteren Grenze der Messergebnisse nicht gut erfasst werden. In der Tat lagen die mittleren ESSDAI-Werte in verschiedenen Langzeitkohorten [28–30] im Bereich von 3–5 Punkten – mit wenig dynamischen Veränderungen im Verlauf.

Zudem ist die Entwicklung des PRO für den ESSPRI [20] wichtig, denn hierbei werden patientenrelevante Beschwerden in den Fokus gerückt und zugleich die Betroffenen gefragt. Hierbei werden in den Einzeldomänen des ESSPRI relativ hohe Werte um 7 von 10 berichtet, wobei Trockenheit und Müdigkeit etwas höher liegen als muskuloskeletale Schmerzen [28–30]. Es ist zu erwarten, dass beide Scores in Zukunft bei Entscheidungen in der medikamentösen Therapie und Verlaufskontrollen hinsichtlich des Erfolgs von interventionellen Therapien eine Rolle spielen.

ESSDAI und ESSPRI sind zwei wichtige Säulen der Outcome-Instrumente der 2. Generation

ESSDAI und ESSPRI stellen zwei wichtige Säulen der Outcome-Instrumente der 2. Generation dar, wobei sie zusammen mit dem Ausmaß der Sicca-Symptomatik und dem PaGA unterschiedlich fakturiert in STAR (Tab. 2) bzw. CRESS (Tab. 3) (Composite of Relevant Endpoints for Sjögren’s Syndrome) eingehen. Beide bedürfen bezüglich einer abschließenden Bewertung aber noch weiterer Überprüfungen.

Die Befunde der Histologie aus der Lippenbiopsie spielt zurzeit zwar für die Diagnosestellung eine wichtige Rolle, allerdings haben immunhistologische Veränderungen in den Speicheldrüsen bislang keinen Eingang in Outcome-Bewertungen gefunden. Nichtsdestoweniger eignet sich ein histologischer Score, der möglicherweise besser quantifiziert ist als der diagnostische Score von Chisholm und Mason [31], möglicherweise als objektivierbarer Parameter für die Einschätzung des Ausmaßes der Entzündung in den Speicheldrüsen [32]. Allerdings ist die Wertigkeit der Assoziation histologischer Befunde zu klinischen Veränderungen und symptomatischer Verbesserung nicht sicher geklärt. Inwieweit sich die Speicheldrüsenentzündung zurückbilden kann und ob Fibrosierung verhindert werden kann, ist ebenfalls noch unklar.

## Fazit für die Praxis

- Es ist davon auszugehen, dass die Sjögren-Erkrankung (Sjögren’s Disease, SjD) in rheumatologischen Praxen über verbesserte Therapiemöglichkeiten wahrscheinlich eine größere Rolle spielen wird.
- Neben der bereits zugelassenen und verfügbaren symptomatischen Therapie, wie künstliche Tränenflüssigkeit, Ciclosporin-A-haltige Augentropfen und die Sekretagoga werden medikamentöse Strategien zur Behandlung der extraglandulären Symptome wahrscheinlich verfügbar werden.
- Zum Verständnis der zugrundeliegenden Studienergebnisse in der Indikationsstellung sind Grundkenntnisse von EULAR Sjögren’s Syndrome Disease Activity Index (ESSDAI) und EULAR Sjögren’s Syndrome Patient Reported Index (ESSPRI) unseres Erachtens wichtig.
- Inwieweit sich diese Instrumente aufgrund ihres Umfangs und ihrer Komplexität für die klinische Praxis eignen, wird sich zeigen.
